# Serum-derived exosomes of young rats protect bone of ovariectomized rats after fatigue loading in vivo

**DOI:** 10.1093/jbmrpl/ziaf164

**Published:** 2025-10-31

**Authors:** Jingqiong Xun, Zhuoyue Lv, Yueming Mei, Meilu Liu, Chan Li, Yuling Liu, Qian He, Bo Wu, Ruchun Dai

**Affiliations:** Department of Endocrinology, Guizhou Provincial People's Hospital, Guiyang, Guizhou, 550002, China; National Clinical Research Center for Metabolic Diseases, Hunan Provincial Key Laboratory of Metabolic Bone Diseases, Department of Metabolism and Endocrinology, The Second Xiangya Hospital of Central South University, Changsha, Hunan, 410125, China; National Clinical Research Center for Metabolic Diseases, Hunan Provincial Key Laboratory of Metabolic Bone Diseases, Department of Metabolism and Endocrinology, The Second Xiangya Hospital of Central South University, Changsha, Hunan, 410125, China; National Clinical Research Center for Metabolic Diseases, Hunan Provincial Key Laboratory of Metabolic Bone Diseases, Department of Metabolism and Endocrinology, The Second Xiangya Hospital of Central South University, Changsha, Hunan, 410125, China; National Clinical Research Center for Metabolic Diseases, Hunan Provincial Key Laboratory of Metabolic Bone Diseases, Department of Metabolism and Endocrinology, The Second Xiangya Hospital of Central South University, Changsha, Hunan, 410125, China; National Clinical Research Center for Metabolic Diseases, Hunan Provincial Key Laboratory of Metabolic Bone Diseases, Department of Metabolism and Endocrinology, The Second Xiangya Hospital of Central South University, Changsha, Hunan, 410125, China; National Clinical Research Center for Metabolic Diseases, Hunan Provincial Key Laboratory of Metabolic Bone Diseases, Department of Metabolism and Endocrinology, The Second Xiangya Hospital of Central South University, Changsha, Hunan, 410125, China; National Clinical Research Center for Metabolic Diseases, Hunan Provincial Key Laboratory of Metabolic Bone Diseases, Department of Metabolism and Endocrinology, The Second Xiangya Hospital of Central South University, Changsha, Hunan, 410125, China; National Clinical Research Center for Metabolic Diseases, Hunan Provincial Key Laboratory of Metabolic Bone Diseases, Department of Metabolism and Endocrinology, The Second Xiangya Hospital of Central South University, Changsha, Hunan, 410125, China; National Clinical Research Center for Metabolic Diseases, Hunan Provincial Key Laboratory of Metabolic Bone Diseases, Department of Metabolism and Endocrinology, The Second Xiangya Hospital of Central South University, Changsha, Hunan, 410125, China

**Keywords:** osteoporosis, exosomes, bone microarchitecture, bone microdamage, bone quality, fatigue loading

## Abstract

In patients with postmenopausal osteoporosis, the accumulation of bone microdamage further increases fracture risk. Exosomes derived from the circulatory system of young individuals can reverse age-related defects during bone repair. Therefore, the present study aimed to elucidate the mechanisms underlying the protective effects of exosomes against structural degradation under fatigue-induced damage. To this end, a rat tibial fatigue injury model was established to investigate the protective effects of serum-derived exosomes (SDEs) isolated from young rats on bone after fatigue damage. SDEs were administered via intramedullary injection for 3 wk. The results demonstrated that treatment with SDEs significantly alleviated bone microdamage in ovariectomized rats. Specifically, it decreased cortical bone microcrack density and increased the mineral apposition rate significantly. In the distal trabecular bone region, treatment with SDEs increased bone volumetric bone mineral density (vBMD) and decreased trabecular spacing (Tb.Sp) significantly, with no significant changes in the structure model index. This study revealed that SDEs can rapidly repair fatigue-damaged bone microstructure, improving microstructural parameters in non-weight-bearing (distal tibial) cancellous bone (increased vBMD and decreased Tb.Sp). These findings provide a potential novel strategy for early intervention of microdamage in postmenopausal osteoporosis.

## Graphical Abstract

**Graphical Abstract:**
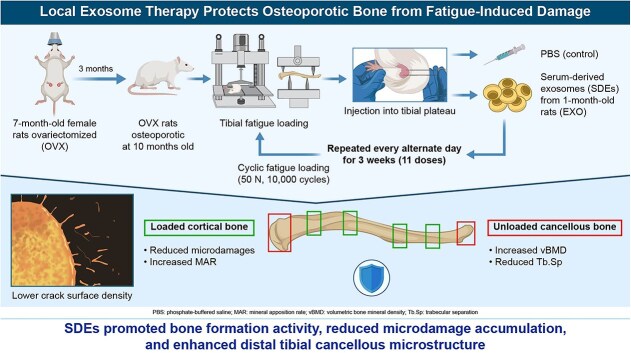

## Introduction

Osteoporosis is a systemic metabolic bone disease characterized by reduced bone mass and impaired bone quality, leading to increased bone fragility and susceptibility to fractures. Osteoporotic fractures, also known as fragility fractures, are the primary cause of disability and mortality in the older adult population and are caused by various factors. Beyond the direct effect of single overload events, such as falls, impact, cyclic fatigue loading during daily activities significantly reduces bone fracture resistance through the progressive accumulation of microdamage.^1–3^ This mechanism is particularly pronounced under pathological conditions of compromised bone quality.^2^ Prospective studies have indicated that approximately 25% of older patients with femoral neck fractures exhibit a prodromal stress fracture phase.^4^ This phase is characterized by the progression of occult bone cracks and progressive limb pain, culminating in a spontaneous fracture due to critical crack propagation, often described by patients as their “legs giving way.”^4^ Notably, the correlation between radiological evidence, such as the detection of stress-induced fractures days before complete fracture, and clinical symptoms supports the notion that bone fatigue may be a significant underlying mechanism of hip fragility fractures in specific patient populations without a history of significant trauma.^5^

Current osteoporosis research models predominantly focus on bone loss and subsequent fracture healing,^6^ and models investigating the repair of microdamage to prevent fractures remain relatively scarce.^7^ In the present study, we applied cyclic bending and loading along the tibial long axis (non-axial compression) of rats to simulate the repetitive mechanical stress encountered by humans during daily activities, inducing linear microdamage on the cortical bone surface. This model closely approximates the physiological load accumulation-induced bone deterioration process observed in osteoporosis.^8^

Bone quality refers to all characteristics other than bone mass that influence the ability of bones to withstand mechanical loads. While changes in bone mass are strongly correlated with fracture risk,^9^^,^^10^ there is a substantial overlap in bone mass between individuals who experience fractures and those who do not. Notably, among individuals aged over 55 yr, over half of all nonvertebral fractures occur in those with clinically normal bone mineral density (BMD).^10^ The limited predictive value of bone mass alone in assessing fracture risk underscores the importance of additional bone quality parameters.^11^^,^^12^

Bone quality is assessed on multiple levels. At the microscale level, encompassing tissue microstructure, compositional turnover, and microdamage homeostasis, compromised bone quality presents as: (1) adaptive deterioration of microstructural integrity, such as reduced cortical thickness and increased total porosity, which significantly diminishes resistance to deformation;^13^^,^^14^ and (2) disruption of microdamage homeostasis, a concept first proposed by Frost in 1960.^14^^,^^15^ This refers to microfractures in the bone matrix, manifesting as linear microcracks and diffuse microdamage,^16^ caused by fatigue loading. Effective repair of such microdamage relies on the tightly regulated balance between bone resorption and formation during remodeling.^17^^,^^18^ However, the bone turnover rate, which cannot distinguish between resorption and formation processes, is limited in its utility for independently evaluating bone quality. In contrast, the mineral apposition rate (MAR), quantified directly via tetracycline double-labeling, specifically measures the rate of bone matrix mineralization. The MAR serves as a key dynamic indicator of osteoblast activity, making it a highly specific and quantifiable proxy for osteoblast-mediated repair capacity.^13^

Exosomes, as vital mediators of intercellular communication, hold a unique potential in regulating bone homeostasis.^19–21^ The low immunogenicity and cargo of diverse bioactive molecules, such as microRNAs (miRNAs), make them potential tools for osteoporosis treatment.^22–24^ Notably, exosomes derived from the circulatory system of young individuals can reverse age-related defects during bone repair.^25^^,^^26^ Moreover, our preliminary experiments demonstrated that serum-derived exosomes (SDEs) of young rats can significantly enhance the osteogenic differentiation capacity of bone marrow mesenchymal stem cells (BMSCs) in ovariectomized (OVX) rats. Therefore, in the present study, we established an OVX rat tibial fatigue loading model to systematically evaluate the protective effects of SDEs obtained from young rats on bone mass. We assessed volumetric BMD (vBMD) and bone quality, including microdamage repair efficiency, microstructural parameters, and bone formation activity. This study aimed to elucidate the mechanisms underlying the protective effects of exosomes against structural degradation caused by fatigue-induced damage, thereby providing theoretical and experimental foundations for exosome-targeted modulation of bone quality to prevent osteoporotic fractures.

## Materials and methods

### Experimental animals

This study utilized the classic osteoporosis model using OVX rats, as their long bone structure supports in vivo fatigue loading and facilitates subsequent hard tissue section preparation. Clean-grade female Sprague-Dawley (SD) rats (*n* = 40) were obtained from the Experimental Animal Center of the Second Xiangya Hospital, Central South University. Of these, 16 1-mo-old females were used exclusively for serum exosome isolation. The remaining 24 7-mo-old female rats (weighing 220 ± 10 g) were maintained under controlled conditions (25 ± 2 °C, 45%-55% humidity, and 12/12 h light/dark cycle) and allocated into three initial groups: (1) Exosome biodistribution group (*n* = 4), (2) Bone validation group (*n* = 16), (3) Reserve group (*n* = 4). Following biodistribution imaging, the biodistribution rats (*n* = 4) and reserve rats (*n* = 4) were combined to form an acute toxicity group (*n* = 8). Detailed experimental procedures for each group are described in section “Experimental design”. All animal procedures were approved by the Animal Care and Ethics Committee of the Second Xiangya Hospital, Central South University (Approval No. 20201220).

### Ovariectomy

The 7-mo-old rats were subjected to ovariectomy. Briefly, the rats were anesthetized with 3% sodium pentobarbital (1 mL/kg), the fallopian tubes were ligated, and both ovaries were removed. OVX rats were aged until 10 mo of age and then used for the fatigue loading experiments.

### Experimental design

Group-specific interventions:(1) Biodistribution group: Rats received intratibial marrow cavity injections of either PBS (*n* = 2) or DiR-labeled exosomes (*n* = 2), followed by in vivo fluorescence imaging at 36 h. (2) Bone validation group: All rats underwent bilateral cyclic tibial fatigue loading every other day for 3 wk to induce microdamage. After fatigue loading, they were randomized into the exosome-treated (*n* = 8) and control (*n* = 8) groups. Starting from the final loading day and continuing every other day thereafter, the exosome-treated group received bilateral intratibial marrow cavity injections of SDEs (1500 μg per tibia), whereas the control group received bilateral injections of an equal volume of PBS into the bone marrow cavity (intramedullary; total volume, including flush, <500 μL per tibia). Three weeks post-final injection, the rats were euthanized for analysis. Specifically, the left tibiae were assessed for microdamage using basic fuchsin and dynamic histomorphometry using calcein/alizarin double-labeling. The right tibiae were subjected to microcomputed tomography (μCT) to assess trabecular and cortical microarchitecture. (3) Acute toxicity group: Rats underwent bilateral tibial fatigue loading (every other day, 2 wk) with concurrent PBS/exosome injections. Major organs were harvested for H&E staining post-euthanasia. No adverse events were observed in any rats during the experimental period.

### Exosome isolation and characterization

SDEs were isolated according to the established protocol described in our previous study,^27^ with minor modifications. Briefly, after anesthetizing the 1-mo-old rats, blood samples were collected from the abdominal aorta, maintained at ambient laboratory temperature (22-26 °C) for 1 h, and then centrifuged at 3000 × *g* for 10 min. Serum was pre-cleared via centrifugation (3000 × *g*, 30 min), diluted with PBS (4:1), and centrifuged at 10 000 × *g* (40 min, 4 °C). The supernatant was concentrated using a 10-kDa ultrafiltration tube (3000 × *g*, 40 min, 4 °C). The filtrates were collected and mixed with the ExoQuick solution (250:63 ratio), incubated (30 min), and then centrifuged (1500 × *g*, 30 min). The pellet was washed with PBS using centrifugation (1500 × *g*, 5 min), and the residual supernatant was completely removed. The final exosome pellet was resuspended in PBS and stored at 4 °C for short-term use or −20 °C for long-term preservation.

Exosome characterization was performed in accordance with the MISEV2018 guidelines^28^ and our previous experimental expertise.^27^ To assess morphology, transmission electron microscopy (TEM) imaging was performed after 2% uranyl acetate staining (FEI Tecnai G2 Spirit Twin). To analyze size distribution, nanoparticle tracking analysis (Particle Metrix Nanosight 2000, 25 °C) was performed. To assess protein markers, western blot analysis of the expression of exosome markers (CD9, 1:1000; TSG101, 1:500; Abcam, Cambridge, UK) was performed.

### Intratibial injection and biodistribution of SDEs

Bilateral intratibial injections targeting the tibial bone marrow cavity were administered through the tibial plateau. For biodistribution assessment, the following PBS-resuspended formulations (total injection volume, including flush, <500 μL per tibia) were administered: 1000 μg of DiR-labeled exosomes (DiR-EXO1 group), 2000 μg of DiR-labeled exosomes (DiR-EXO2 group), 2000 of μg unlabeled exosomes (EXO group), and 2000 μg of free DiR dye (DiR group). Biodistribution was monitored at 16 and 36 h post-injection using an in vivo fluorescence tomography imaging system (PerkinElmer FMT4000; Waltham, MA, USA). For therapeutic evaluation, 1500 μg exosomes were injected per tibia (SDEs group; *n* = 8) and or an equal volume of PBS (control group; *n* = 8).

### Fatigue loading

Fatigue loading was applied to induce microdamage. Following anesthesia, a four-point bending fatigue loading test was performed using an electronic fatigue machine (Chinese Patent No. ZL00225310.0). Cyclic fatigue loading was performed on the bilateral tibiae of rats in the loading group under anesthesia at a peak force of 50 N and a valley force of 40 N (sine wave: 4 Hz, 10 000 cycles). The bearing force points are marked. The mechanical loading parameters were determined based on tibial load data from 22-mo-old OVX rats,^27^ where the ultimate load (Fmax) was 85.6 ± 12.7 N. Consistent with the reported age-related decline in bone biomechanical properties,^29^ the ultimate load in 22-mo-old rats decreased by approximately 10%-15% compared with that in 10-mo-old rats. Thus, the projected ultimate load for 10-mo-old rats was extrapolated as follows: Fmax (10 mo) = Fmax (22 mo)/(1 − η) (η = 0.10-0.15). The experimental load was subsequently set at 40%-60% of the projected ultimate load for 10-mo-old rats (38.05-57.08 N). This range effectively induced bone microdamage while preventing acute fractures, as validated by the measured values at the corresponding percentages in the 22-mo-old rats. Mechanical loading was applied to the rats using an electronic four-point bending fatigue-testing machine. The spatial configuration of the force application points is detailed in Supplementary File 1: Figure S1 of our previous study.^27^

### H&E staining

After exosome injection and fatigue loading, the rats were euthanized, and their hearts, livers, and kidneys were fixed and embedded in paraffin, sectioned, and stained with H&E to observe the acute toxic effects of treatment on vital organs. The liver, heart, and kidney samples were fixed in 4% paraformaldehyde for 24 h at 4 °C and embedded in paraffin blocks. These blocks were further sectioned at 5 μm, stained with H&E, and then examined under a microscope (SkyScan-1176; Bruker, Billerica, MA, USA).

### Bone microdamage evaluation

The tibiae were subjected to alkaline fuchsin staining, plastic-embedding, hard tissue sectioning, light microscopy examination, and bone microdamage evaluation.^30^^,^^31^ Briefly, the tibiae were dehydrated and stained in sublimated alcohol containing 1% alkaline fuchsin, subjected to hyalinization and dimethylbenzene treatment, and embedded in polymethyl methacrylate until polymerization was complete. Thick sections (80-100 μm) were cut transversely and sequentially at stress points with a diamond saw and observed under a Leica DMLA polarized light microscope (Leica, Wetzlar, Germany). Images of areas with microdamage were captured. A Leica Qwin (Leica) was used to measure the microdamage parameters, including the microcrack length, number of microcracks, microcrack surface density, and microcrack density. Data were collected at four locations on each tibia. The same sections were subsequently used for calcein and tetracycline fluorescence labeling, as described in section “Calcein and tetracycline fluorescence labeling and MAR evaluation”, and the tibiae were processed under minimal light exposure.

### Calcein and tetracycline fluorescence labeling and MAR evaluation

The rats were injected intraperitoneally with tetracycline (30 mg/kg body weight) 21 and 20 d before euthanasia, and intraperitoneally with calcein (5 mg/kg body weight) 4 and 3 d before euthanasia. Following euthanasia, the tibiae of the rats were removed, embedded in plastic, and subjected to hard tissue dissection. The sections were observed under a fluorescence microscope. The distance between the bright-green and orange-yellow fluorescent lines represents the amount of bone formation between injections. Qwin image analysis software was used to measure the spacing between the fluorescent lines of tetracycline and calcein. The MAR, measured as the spacing/number of days between dosing times (17 d), was calculated. Statistical analyses were performed using GraphPad Prism (GraphPad Inc., La Jolla, CA, USA) software.

### μCT analysis

Rat tibiae were scanned using a SkyScan-1176 μCT scanner (Bruker micro-CT, Bruker). The scanner voltage and current were set to 80 kV and 278 μA, respectively, with a resolution of 18 μm per pixel. NRecon (v1.6) and CTan (v1.13) software were used for image reconstruction and analysis, respectively. Four cortical bone ROIs, each spanning 2-mm in thickness, were analyzed at loading sites symmetrically distributed around the tibial midpoint: 2 proximal sites positioned 3-5 mm and 8-10 mm from the midpoint and two distal sites at equivalent distances (3-5 mm and 8-10 mm distally), with all spatial parameters defined. The ROIs for cancellous bone selected for analysis included 224 layers (4 mm) starting 1 mm below the growth plate of the tibial plateau, and 140 layers (2.5 mm) starting 1 mm above the growth plate of the distal tibia (ankle).

### Statistical analysis

Data analysis was performed using SPSS 19.0 software (SPSS Inc., San Diego, CA, USA), with a significance level set at α = .05 (two-tailed test). Normality testing was conducted for all data using the Shapiro–Wilk test to evaluate the distribution patterns of two independent samples (*n* = 8 per group). A group was considered approximately normally distributed if *p* > .05, and non-normally distributed if *p* ≤ .05. For non-normally distributed data (where at least one group had *p* ≤ .05), the Mann-Whitney U test was used to compare distribution differences between groups. For normally distributed data (*p* > .05 in both groups), Levene’s test for homogeneity of variance was further performed. When variances were homogeneous (Levene *p* > .05), an independent samples *t*-test (Student’s *t-*test) was used to analyze mean differences. When variances were heterogeneous (Levene *p* ≤ .05), the Welch-Satterthwaite corrected *t*-test was applied to account for variance heterogeneity and maintain test power. Statistical significance was defined as *p* < .05. Data are presented as mean ± standard deviation (M ± SD) or median (interquartile range, M [P_25_, P_75_]). Normally distributed data were visualized using bar charts with error bars (error bars represent SD), whereas non-normally distributed data were displayed using box plots to depict distribution characteristics (including median and quartiles).

## Results

### Characterization of SDEs

**Figure 1:**
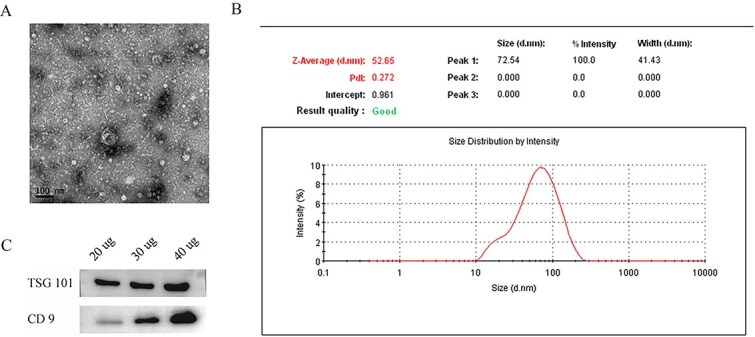
Characterization of SDEs. (A) Saucer-like structures of SDEs were observed using transmission electron microscopy. Scale bar, 100 nm. (B) Western blotting of the expression of the marker proteins CD9 and TSG101in the SDEs. (C) Particle size distribution of SDEs. Abbreviation: SDEs, serum-derived exosomes.

We characterized the exosomes using TEM, which revealed a disc-shaped morphology with a diameter of approximately 50-100 nm, corresponding to the typical structure of exosomes (Figure 1A). In addition, diffusion light scattering measurements demonstrated a single peak with a diameter of approximately 80 nm (Figure 1B). These results confirm the identity of the SDEs. We confirmed the expression of the exosome marker proteins CD9 and TSG101 using western blotting, which revealed strong bands of CD9 and TSG101 in exosome-rich particles (Figure 1C).

### SDE distribution following their injections into the bone marrow cavity

**Figure 2:**
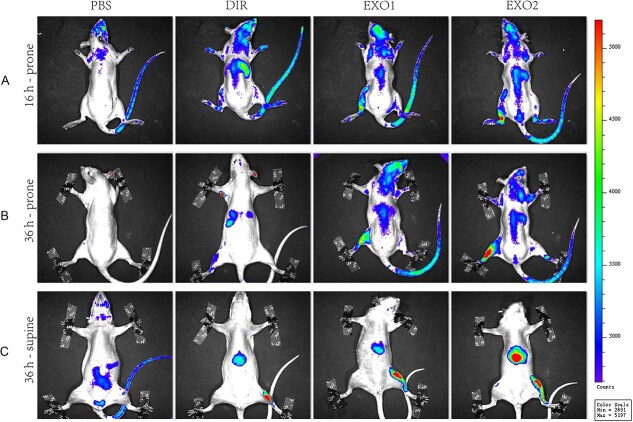
Bone marrow injection improves SDE accumulation in the bone. After injecting SDEs into the tibial bone marrow cavity of rats in the SDE injection (SDE) and DiR dye injection groups (DiR), DiR-labeled different concentrations of SDEs (DiR-SDEs 1 and DiR-SDEs 2) were imaged in the prone position at 16 h (16 h-prone) and 36 h of injection (36 h-prone), as well as in the supine position (36 h-supine), for fluorescence imaging. Abbreviation: SDEs, serum-derived exosomes.

To increase the local concentration of SDEs in the bone marrow cavity and avoid their dilution in the circulation, which would weaken their effect on tibial microdamage, we injected DiR-labeled SDEs into the bone marrow cavity and performed fluorescence imaging at 16 and 36 h to observe the distribution of SDEs in rats. We observed local fluorescence signals in the bone marrow cavity 16 h after the injection of SDEs into the left tibial bone marrow cavity. At 36 h, we observed increased fluorescence accumulation in the liver, while local fluorescence concentrations of exosomes remained detectable in the bone marrow cavity (Figure 2C).

### Treatment with SDEs exhibits no toxicity to tissues following injection into the bone marrow

We injected SDEs derived from young rats into the tibial bone marrow cavity of aged osteoporotic rats subjected to fatigue loading, every alternate day for 2 wk. Control mice received PBS injections into the tibial marrow cavity. Following euthanasia, the hearts, livers, and kidneys of rats were sectioned and stained with H&E to assess the acute toxic effects of SDEs on the vital organs. Microscopic analysis revealed that the liver lobules in the SDE group were structurally intact and clearly outlined, with a small number of fat vacuoles, and no significant increase in fat vacuoles compared with the PBS group. The longitudinal section of the myocardium in the SDE group exhibited a slightly slender and blurred pattern of myocardial cell arrangement, with a tendency for the cells to fuse. However, there was no significant difference in the pattern between the groups, and the difference between the SDE and PBS groups was also not significant (Figure S1).

### Treatment with SDEs derived from young rats promotes tibial bone formation in osteoporotic rats subjected to fatigue loading

**Figure 3:**
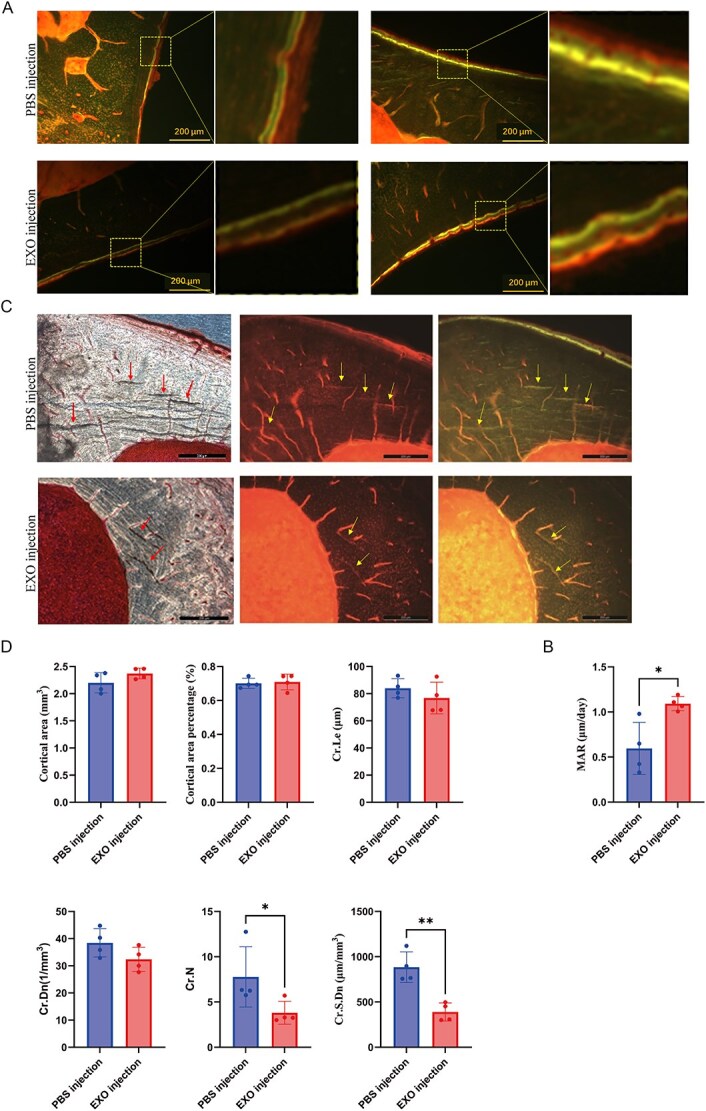
Treatment with SDEs derived from young rats mitigates bone microdamage caused by fatigue loading in the tibiae of osteoporotic rats. (A, B) Bone mineralization rate (MAR) of the tibiae of rats with fatigue loading-induced osteoporosis injected with either PBS (PBS injection group) or SDEs from young rats (EXO group). (A) Representative fluorescence microscopy images showing sequential double-labeling with calcein (first label) and tetracycline (second label) to measure the mineral apposition rate. (B) Quantification of the mineral apposition rate (MAR) value. (C, D) bone microdamage in the EXO and PBS groups. (C) Representative images of bone microdamage (scale bar: 200 μm). (D) Quantitative assessment of morphological parameters of microdamage in the cortical area, including the percentage of damaged area, microcrack length (Cr. Le), microcrack density (Cr. Dn), microcrack number (Cr. N), and microcrack surface density (Cr. S. Dn). ^*^p < .05, ^**^p < .01, *n* = 4. Data are presented as mean ± standard deviation. Abbreviations: PBS, phosphate buffer saline; MAR, mineral apposition rate.

Tetracycline and calcineurin specifically bind to osteocalcin and are deposited at the mineralization front during bone formation. Therefore, we administered tetracycline and calcineurin to rats at different times to distinguish the temporal dynamics of bone formation. The SDEs were injected into the tibial bone marrow cavity of osteoporotic rats subjected to fatigue loading for 21 d. Two injections of tetracycline were administered on days 1 and 2 at the beginning of the intervention, while two injections of calcineurin were administered on days 4 and 3 before the intervention was completed. The results indicated that fluorescence line distance was greater in the SDE group than in the PBS injection group (Figure 3A). Moreover, the bone MAR was higher in the SDE group than in the PBS injection group (Figure 3B). These results indicate that SDE injections promoted tibial bone formation compared to the PBS group.

### Treatment with SDEs derived from young rats mitigates bone microdamage caused by fatigue loading in the tibiae of osteoporotic rats

After intramedullary injections of the SDEs into the tibiae of osteoporotic rats subjected to fatigue loading, we observed microdamage under a microscope (Figure 3C). Red fluorescence refractive lines were visible under red light, while green fluorescence refractive lines were visible under green light in the corresponding parts. In contrast, cracks introduced artificially during later hard tissue sectioning did not exhibit fluorescence.

We further analyzed morphological parameters of bone microdamage, including cortical area, cortical area percentage, microcrack length, microcrack density, microcrack number, and surface density. The cortical area and its percentage did not significantly differ between the two groups. The microcrack length in the SDE injection group exhibited a decreasing but non-significant trend. The microcrack number and surface density were significantly lower in the SDE group than in the control group, indicating a decreased degree of microdamage (Figure 3D). These results indicate that SDE injections significantly reduced bone microdamage compared with PBS injections.

### Treatment with SDEs derived from young rats does not significantly affect the cortical bone microarchitecture of osteoporotic rats with bone microdamage

**Figure 4:**
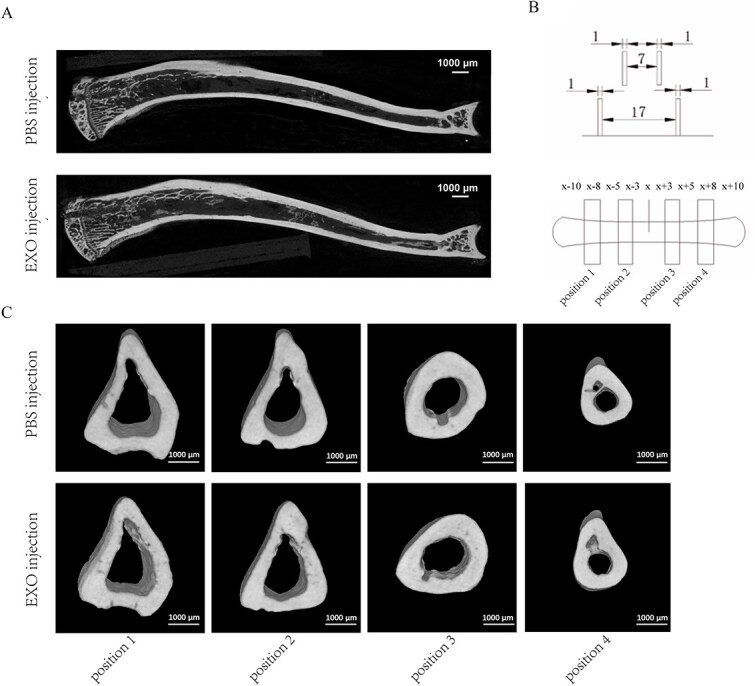
Improvement of bone microstructure in rats with bone microdamage following injection with SDEs derived from young rats. (A, C) three-dimensional reconstruction of bone microstructure in coronal and longitudinal tibia. (B) Illustration of fatigue loading point and μCT ROI in rat tibia. Positions 1, 2, 3, and 4 are proximal and distal to the X-point. For tibial cortical bone scan analysis, 3-5 mm and 8-10 mm were used as the ROIs. Abbreviations: SDEs, serum-derived exosomes; μCT, microcomputed tomography.

After the 3-wk intervention, the rats were euthanized, and the tibiae were subjected to μCT scanning to assess bone microarchitecture examination of both cortical and cancellous bone in the proximal and distal regions. The X-axis position was centered at the midpoint of the loading site on both tibiae. Four analysis positions, designated as positions 1, 2, 3, and 4, were sequentially defined from the proximal to the distal end of the tibia relative to the fatigue loading site (Figure 4B). Three-dimensional reconstruction of the bone microarchitecture in the SDE and PBS injection groups in the sagittal (Figure 4A) and transverse sections at the four loading positions of the tibiae (Figure 4C). The cortical bone microarchitectural parameters at positions 1, 2, 3, and 4 were not statistically different between the SDE and PBS injection groups (*p* > .05; Table 1), including vBMD, BV/TV and cortical bone thickness. These results confirm that injections of SDEs derived from young rats exhibited no significant effect on the cortical bone microarchitecture.

**Table 1:** Comparison of cortical bone microarchitecture parameters between the PBS and EXO injection groups.

**Position**	**Var.** ^**a**^	**Norm.** ^**b**^	**PBS injection**	**EXO injection**	***p*-Value**	**Statistical test** ^**c**^
**Position 1**	vBMD (g/cm^3^)	N	0.4289 ± 0.0172	0.4323 ± 0.0076	.6212	Welch’s *t-*test
	BV/TV (%)	NN	83.00 (1.65)	83.60 (1.04)	.1605	Mann–Whitney U
	Cor.Th (mm)	NN	0.6386 (0.0719)	0.6489 (0.0198)	.4418	Mann–Whitney U
**Position 2**	vBMD (g/cm^3^)	N	0.4348 ± 0.0122	0.4394 ± 0.0104	.4331	Student’s *t*-test
	BV/TV (%)	N	83.47 ± 0.6110	84.08 ± 0.9135	.1621	Student’s *t*-test
	Cor.Th (mm)	N	0.6582 ± 0.0228	0.6777 ± 0.0271	.1416	Student’s *t*-test
**Position 3**	vBMD (g/cm^3^)	N	0.4708 ± 0.0180	0.4697 ± 0.0076	.8765	Student’s *t*-test
	BV/TV (%)	N	85.90 ± 0.5872	85.90 ± 0.4132	.9988	Student’s *t*-test
	Cor.Th (mm)	N	0.7293 ± 0.0338	0.7289 ± 0.0180	.9738	Student’s *t*-test
**Position 4**	vBMD (g/cm^3^)	N	0.4470 ± 0.0117	0.4483 ± 0.0130	.8262	Welch’s *t*-test
	BV/TV (%)	N	85.66 ± 1.611	86.39 ± 1.332	.3593	Welch’s *t*-test
	Cor.Th (mm)	NN	0.7545 (0.0563)	0.7584 (0.0418)	.7209	Mann–Whitney U

Data are presented as mean ± SD or median (IQR) based on normality There were no significant differences in volumetric bone mineral density (vBMD), bone volume percentage (BV/TV), or cortical bone thickness for each ROI in the EXO group compared with the PBS group (*p* > .05; *n* = 8). ^a^Var., variable definitions; vBMD, volumetric bone mineral density; BV/TV, bone volume fraction; Cor.Th, cortical thickness. ^b^Norm., normality tested using the Shapiro–Wilk test (N: normally distributed, *p* > .05; NN: non-normal, *p* ≤ .05). ^c^Statistical tests were selected based on the normality of data distribution (as indicated in the Norm. column).

### SDE injections ameliorate the microstructural properties of tibial trabecular bone

**Figure 5:**
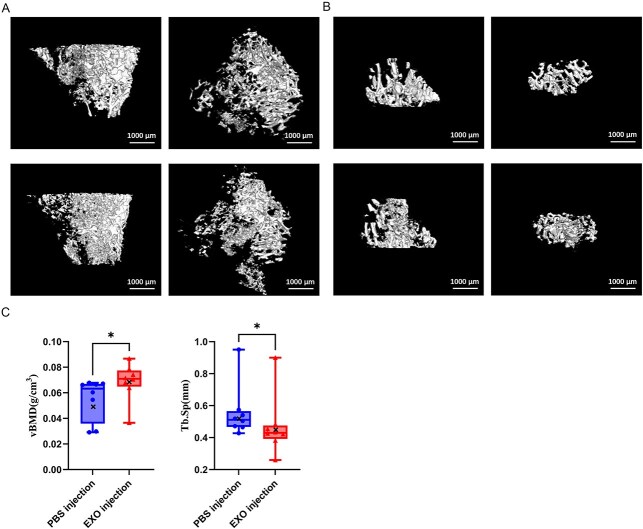
Effects of SDEs on tibial cancellous bone microarchitecture. (A, B) three-dimensional μCT reconstruction of proximal and distal tibial cancellous bone (coronal and horizontal views) showing trabecular structural changes following PBS or SDE injections. (C) Quantitative analysis of distal tibial cancellous bone parameters. Data are presented as mean ± SD (*n* = 8). ^*^p < .05. Abbreviation: SDEs, serum-derived exosomes.

Our μCT analysis demonstrated region-specific responses to SDE injections in tibial cancellous bone (Figure 5, Table 2). The proximal tibia exhibited increasing but insignificant trends in vBMD and BV/TV (both *p* > .05). Improvements were observed in the distal region, where vBMD significantly increased (*p* = .0207) and trabecular spacing decreased (*p* = .0379). Notably, both regions maintained stable trabecular thickness (Tb.Th) and structural model index (all *p* > .5), suggesting that treatment SDEs may exert a more pronounced effect on trabecular spatial organization than overall structural remodeling.

**Table 2:** Comparison of cancellous bone microarchitecture parameters between the PBS and EXO injection groups.

**Position**	**Var.** ^**a**^	**Norm.** ^**b**^	**PBS injection**	**EXO injection**	***p*-Value**	**Statistical test**
**Distal**	vBMD (g/cm3)	NN	0.06328 (0.03097)	0.07095(0.01258)	.0207^*^	Mann–Whitney U
	BV/TV (%)	NN	13.52 (4.49)	15.56 (4.07)	.0830	Mann–Whitney U
	Tb.Th (mm)	N	0.1019 ± 0.0027	0.0995 ± 0.0058	.3080	Student’s *t*-test
	Tb.Sp (mm)	NN	0.5114 (0.0987)	0.4296 (0.0835)	.0379^*^	Mann–Whitney U
	Tb.N (1/mm)	N	1.254 ± 0.2355	1.473 ± 0.2789	.1118	Student’s *t*-test
	SMI	N	2.239 ± 0.1945	2.279 ± 0.2209	.7008	Student’s *t*-test
	Conn.Dn (1/mm3)	N	0.0001 ± 3.314 × 10^5^	0.0002 ± 7.240× 10^5^	.1137	Student’s *t*-test
**Proximal**	vBMD (g/cm3)	N	0.0545 ± 0.0153	0.0707 ± 0.0213	.1031	Student’s *t*-test
	BV/TV (%)	N	9.202 ± 2.372	11.54 ± 3.937	.1718	Student’s *t*-test
	Tb.Th (mm)	N	0.0908 ± 0.0063	0.09921 ± 0.0061	.6826	Student’s *t*-test
	Tb.Sp (mm)	NN	0.5191 (0.1583)	0.3968 (0.2009)	.2786	Mann–Whitney U
	Tb.N (1/mm)	NN	0.9997 (0.4149)	1.207 (0.045)	.1605	Mann–Whitney U
	SMI	N	2.510 ± 0.2844	2.494 ± 0.3019	.9175	Student’s *t*-test
	Conn.Dn (1/mm3)	N	0.0001 ± 5.600 × 10^5^	0.0002 ± 8.975 × 10^5^	.2616	Student’s *t*-test

Data presented as mean ± standard deviation or median (IQR) based on normality; *n* = 8. ^a^Var., variable definitions: vBMD, volumetric bone mineral density; BV/TV, bone volume fraction; Tb.Th, trabecular thickness; Tb.Sp, trabecular spacing; Tb. N, trabecular number; SMI, Structure Model Index; Conn. Dn, trabecular connectivity density. ^b^Norm., normality tested via Shapiro–Wilk test (N: normally distributed, *p* > .05; NN: non-normal, *p* ≤ .05). ^*^ *p* < .05.

## Discussion

### Model establishment

This study investigated the multilevel effects on bone tissue of a short-term (3-wk) intervention using local intramedullary injection of SDEs derived from young rats. Our preliminary experiments involving tail vein injection showed no significant exosomal enrichment in the tibia (Figure S2), a finding consistent with the reported rapid systemic clearance of intravenously administered exosomes.^32^ These results supported the adoption of a local intramedullary injection strategy to enhance site-specific delivery.

To validate this strategy, in vivo imaging was performed after local tibial injection of DiR-labeled SDEs, which confirmed their sustained enrichment within the tibia. As demonstrated in Figure 2, strong fluorescence was highly localized to the injection site at the 16-h time point. By 36 h, while pronounced signal persisted in the tibia, fluorescence also emerged in the abdominal region—indicating a transition from initial local retention to gradual systemic release. This spatiotemporal pattern aligns with the typical clearance of extracellular vesicles via the mononuclear phagocyte system.^33^ Importantly, despite observed off-target signals, H&E staining revealed no significant histopathological alterations or acute toxicity in major organs, supporting the preliminary safety of this delivery approach.

### Microscale effects of SDEs on bone formation and microdamage repair

Age-related osteoporotic fractures are associated with microdamage accumulation,^34^ with linear microcracks (20-200 μm) representing the primary pathological form.^14^ In the present study, short-term local medullary injection of young rat SDEs significantly reduced tibial microdamage density. Morphological analysis revealed that SDE injection decreased the microcrack number but did not affect the microcrack length. Notably, this rapid reduction in microcrack number observed contrasts with classical bone remodeling, which is dependent on osteoclast-osteoblast cycles and typically requires longer timelines, although SDEs enhanced osteogenic activity. This is consistent with prior evidence that SDEs promote BMSC osteogenic differentiation.^27^ We propose two potential early repair mechanisms: (1) SDEs may induce osteoclasts to clear small, repairable microcracks (≤50 μm) and directly initiate local mineralization; (2) osteocytes, functioning as mechanosensors, may also promote calcium and phosphorus deposition through the regulation of matrix proteins, such as by inhibiting DMP-1,^35^ thereby enabling rapid microcrack repair without classical remodeling. However, our current observations of reduced crack number and stable length are insufficient to determine the predominant mechanism. Future studies are required to dynamically monitor microdamage turnover, incorporating cell tracing, such as osteoclast and osteocyte markers, and molecular assays targeting proteins such as DMP-1 and mineralization-related factors. These approaches will help elucidate the early repair network mediated by SDEs and validate their potential novel mechanism.

### Effects of SDEs on trabecular bone microarchitecture

Bone microstructure, particularly the 3-dimensional trabecular network in cancellous bone, serves as a critical structural foundation for resisting microdamage accumulation and maintaining skeletal integrity.^36^^,^^37^ Cancellous bone is characterized by a high surface-area-to-volume ratio which provides an efficient interface for converting mineralization precursors into hydroxyapatite crystals, and proximity to the bone marrow microenvironment. It plays a crucial role in stress buffering, load distribution, and early-stage microdamage inhibition.

#### Microstructural optimization and mechanistic insights of distal tibial cancellous bone

In the present study, the distal tibial cancellous bone exhibited significant microstructural optimization following SDE injections. Specifically, vBMD increased, trabecular spacing decreased, while the structure model index remained unchanged, indicating preservation of fundamental trabecular morphology, such as plate-like versus rod-like distribution. Given that SDE injections did not affect the bone trabecular number and thickness, the microstructural improvements are likely attributed to SDE-mediated accelerated surface mineralization rather than de novo bone formation.

In contrast, literature-reported SDEs derived from young humans enhance bone microenvironmental mineralization efficacy through the co-delivery of enriched miR-142-5p, which simultaneously activates alkaline phosphatase expression and inhibits the osteogenic repressor ZFPM2, ultimately achieving trabecular gap filling, vBMD elevation, and increasing Tb.Th and bone volume fraction (BV/TV).^38^ Pathological adaptability differences in exosomal miRNA profiles have been confirmed to possess specific functions in regulating bone metabolism and promoting bone mineralization.^38^^,^^39^ The optimization of bone microstructure by SDEs in this study may be associated with the mineralization network mediated by such miRNAs, though the specific molecular mechanisms require further validation.

Notably, the differences in the effects of the 2 types of exosomes may be closely related to the pathological characteristics of the models: in the osteoporosis combined with bone microdamage model of this study, SDEs (from young rats) may primarily repair the local microenvironment through targeted bone surface mineralization (eg, ordered collagen deposition, hydroxyapatite crystal formation); whereas in the simple OVX model characterized by excessive bone resorption, SDEs derived from young humans adapt to this pathological demand through systematic osteogenic mechanisms.

#### Limited improvement in proximal trabecular bone

In the SDE injection group, the proximal tibial trabecular bone displayed only trends toward improved microstructure, none of which reached statistical significance. This spatial heterogeneity was primarily attributed to mechanical disruption caused by medullary cavity injection. Specifically, the puncture needle directly damaged the proximal trabecular microstructure, as evidenced by microfractures and disruption of extracellular matrix continuity, creating physical barriers. These barriers, in turn, reduced the distribution efficiency of SDEs and hindered their interaction with target cells, such as bone-lining cells. Consequently, the surface mineralization process, critical for microstructural optimization, was less effective in this region.

### Effects of SDEs on cortical bone structure

Cortical bone loss manifests as marrow cavity expansion, cortical thinning, increased cortical porosity, and reduced vBMD,^40^ collectively compromising fracture resistance. In the present study, μCT analysis of fatigue-loaded cortical sites revealed that although intervention with young rat SDEs significantly reduced local microcrack density, improvements in macrostructural parameters, including vBMD, cortical thickness, BV/TV, and porosity, failed to reach conventional levels of statistical significance within the 3-wk intervention period.

### Cancellous-cortical bone response discordance: possible origins

The phenomenon observed in the present study, characterized by unchanged cortical macrostructure despite cancellous improvement, can be explained by multiple factors. First, the inherent biological characteristics of cortical bone are the basis for its slow response, as its annual renewal rate is considerably slower than that of cancellous bone. Thus, although SDEs increase the local MAR and promote microcrack repair (as evidenced by decreased microcrack density), short-term intervention may not be sufficient to drive significant macroscopic structural changes. Second, the targeted distribution characteristics of SDEs limit their effective enrichment in cortical bone. After intramedullary injection, SDEs preferentially accumulated in cancellous bone regions rich in bone marrow. This resulted in insufficient concentrations of SDEs in cortical bone, especially in areas distant from the medullary cavity, thereby limiting their capacity to support adequate structural repair in these regions. Moreover, the combination of short-term fatigue loading with SDEs intervention in the present study may not have reached the required intervention duration to trigger significant macroscopic remodeling of the cortical bone. Despite differences in study design, a previous study has demonstrated inconsistent responses between cortical and cancellous bone.^41^

However, the rat model exhibits fundamental limitations in simulating human cortical bone pathology.^42^ Owing to the absence of a functional Haversian system in rats, the cortical bone lesions and repair mechanisms in rats are fundamentally different from those in humans. Therefore, the observation of no significant or potential future effects of SDEs on rat cortical bone in this study cannot be directly extrapolated to human cortical bone, especially when predicting its impact on the Haversian remodeling process in humans. In contrast, the high metabolic conservation and structural homology of cancellous bone make the positive response of distal cancellous bone more translationally valuable.

### Research limitations and future directions

This study has certain following limitations. First, the absence of a tail vein injection control group limits quantitative comparison of targeting efficiency, and the local injection procedure may cause minor tissue interference in the proximal tibial region, potentially affecting result interpretation. Future studies should: (1) optimize injection techniques to minimize procedural effects; (2) include both sham and tail vein injection control groups; and (3) employ quantitative biodistribution analyses using exosome-specific markers such as quantitative PCR to differentiate between intervention-related effects and procedure-induced influences.

Second, it lacked biomechanical performance assessment using indicators such as maximum load and stiffness. This prevents confirmation of whether the observed microstructural improvements (especially in the distal cancellous bone) and the reduction in microdamage ultimately translate into a substantial increase in fracture resistance. Future research should incorporate in vitro mechanical tests, such as three-point bending and compression tests, or improve in vivo biomechanical assessment methods.

Third, the intervention period was relatively short. Our study focused on the early-stage microdamage repair phase and did not assess the long-term effects of SDEs on late-stage bone remodeling and structural recovery. The intervention duration limited the evaluation of both the macrostructural effects of SDEs on cortical bone, which may require longer periods to manifest, and their potential role in bone reconstruction. Additionally, while phenotypic microstructural changes, such as vBMD and trabecular spacing, were analyzed, core mechanisms, such as those underlying SDE-mediated surface mineralization acceleration and microdamage feedforward blockade were not validated using mechanistic studies. Therefore, future research should extend the intervention period and integrate mechanistic investigations to better understand the therapeutic potential of SDEs in bone repair.

## Conclusion

In the present, we adopted a novel approach of local bone marrow injection. We revealed, for the first time, the effects of short-term SDE-based intervention in OVX rats with fatigue-induced microdamage. Although cortical bone macrostructural improvements were limited, SDEs significantly promoted bone formation, reduced microdamage accumulation, and optimized distal tibial cancellous microstructure, particularly through optimized trabecular architecture. These findings highlight the potential of SDEs as a rapid-response strategy for mitigating early bone microdamage in osteoporosis.

## Supplementary Material

Supplementary_Fig_ziaf164

## Data Availability

The data supporting the findings of this study are available within the article.
