# Supplementary figures and images for: Serum-derived exosomes of young rats protect bone of ovariectomized rats after fatigue loading in vivo
Source: JBMR Plus. 2025 Oct 31;9(12):ziaf164. doi: 10.1093/jbmrpl/ziaf164 (PMC12646256; doi:10.1093/jbmrpl/ziaf164)

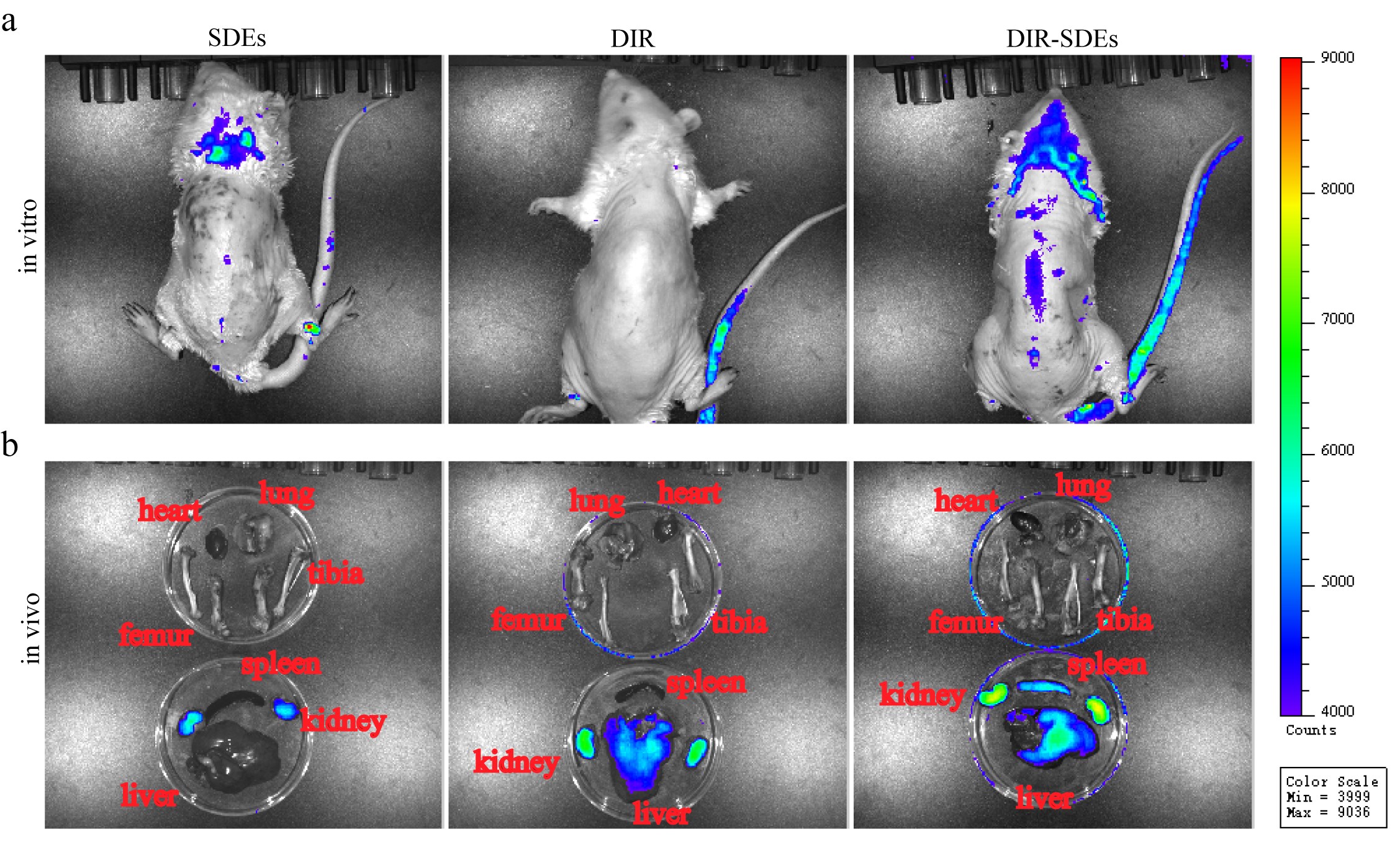

Supplement: Supplementary_Fig_ziaf164 [file supplementary_fig_ziaf164.jpeg]
